# Supplementary material for: Biallelic PAX7 variants cause a novel Satellite Cell-opathy with progressive muscle involvement resembling facioscapulohumeral muscular dystrophy
Source: Cell Death Dis. 2026 Jan 29;17(1):179. doi: 10.1038/s41419-025-08358-6 (PMC12876871; doi:10.1038/s41419-025-08358-6)
Supplement: Supplementary file 1 — Supplementary Information [file 41419_2025_8358_MOESM1_ESM.pdf]

Biallelic *PAX7* variants cause a novel Satellite Cell-opathy with progressive muscle involvement resembling facioscapulohumeral muscular dystrophy

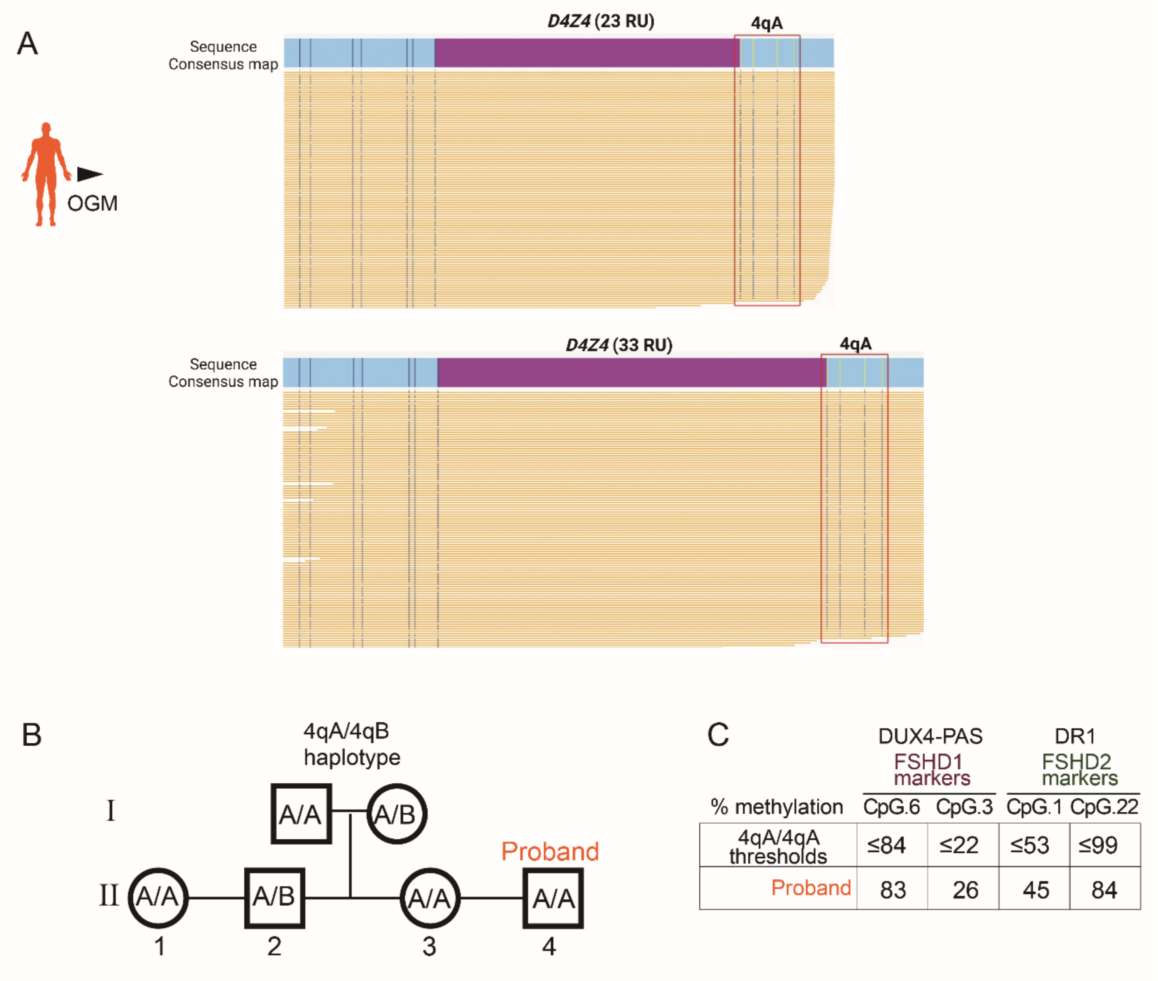

Supplementary Figure 1. **Genetic tests do not indicate FSHD in the proband**
A. Schematic of Optical Genome Mapping (OGM) results on the proband showing the haplotype and the *D4Z4* alleles, excluding in-cis *D4Z4* duplications and mosaicisms.
B. Family pedigree showing 4qA/4qB haplotype in proband and family members.
C. Quantification of methylation levels (%) in proband for FSHD1 (DUX4-PAS; CpG.6 and CpG.3) and FSHD2 (DR1; CpG.1 and CpG.22) markers. Average methylation across the general FSHD population (4qA/4qA) at specific markers is reported for comparison.

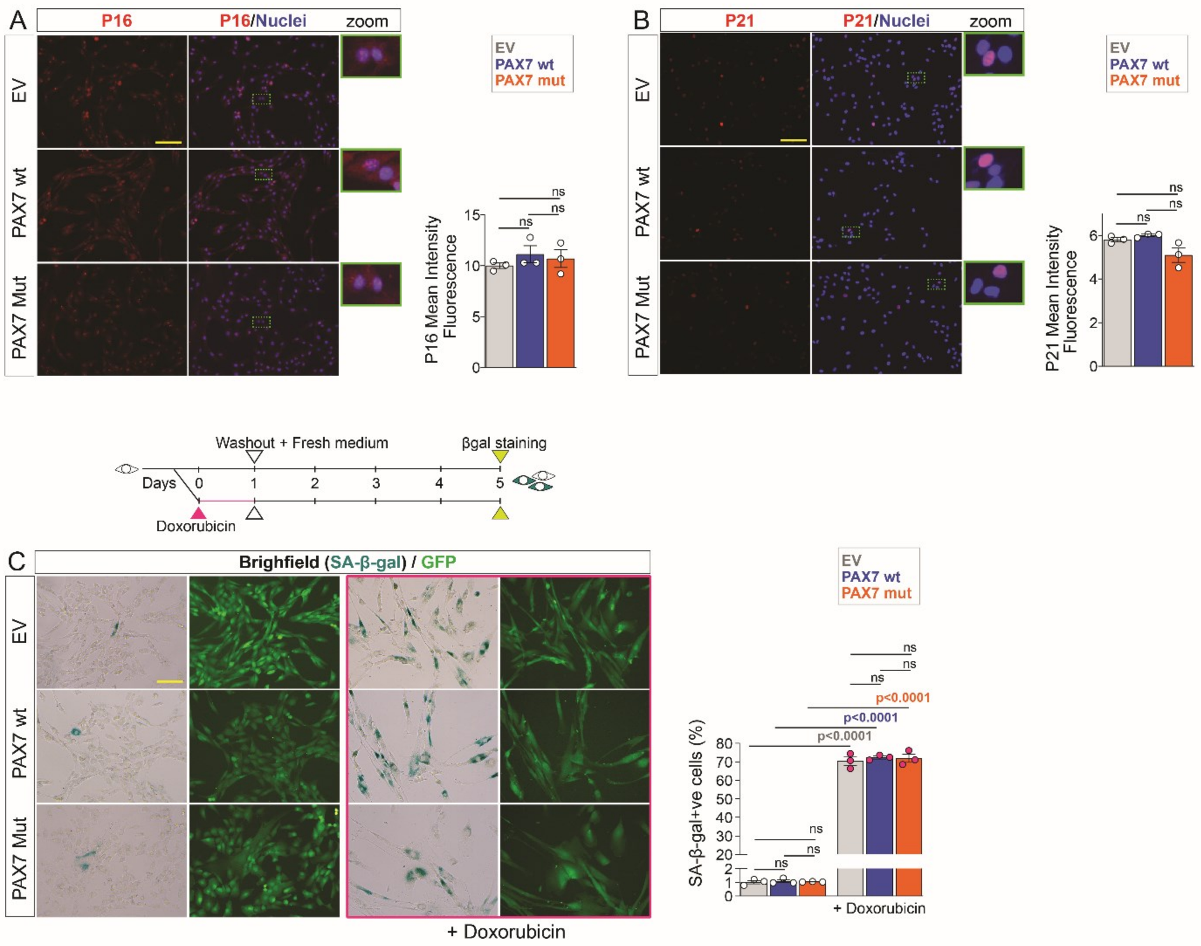

Supplementary Figure 2. **PAX7 P112L-C443Y does not induce or impair cellular senescence.**

A. Representative images of immunolabelling for P16 (red), together with nuclear counterstain (blue) and quantification of fraction of P16+ve myoblasts after 4 days in proliferating conditions show no difference between PAX7wt, PAX7mut and EV cell lines.

B. Representative images of immunolabelling for P21 (red), together with nuclear counterstain (blue) and quantification of fraction of P21+ve myoblasts after 4 days in proliferating conditions show no difference between PAX7wt, PAX7mut and EV cell lines.

C. Schematic of experimental design for senescence analysis. Cells were plated and treated the following day with or without Doxorubicin for 24 hours and, after washout, cultured for 4 days before being fixed for analysis. Representative images of myoblasts assayed for β-gal activity showing increased signal upon Doxorubicin treatment. Quantification shows no difference among the three myoblast populations. All graphs report unpaired t-test analysis, scale bars 100 μm.

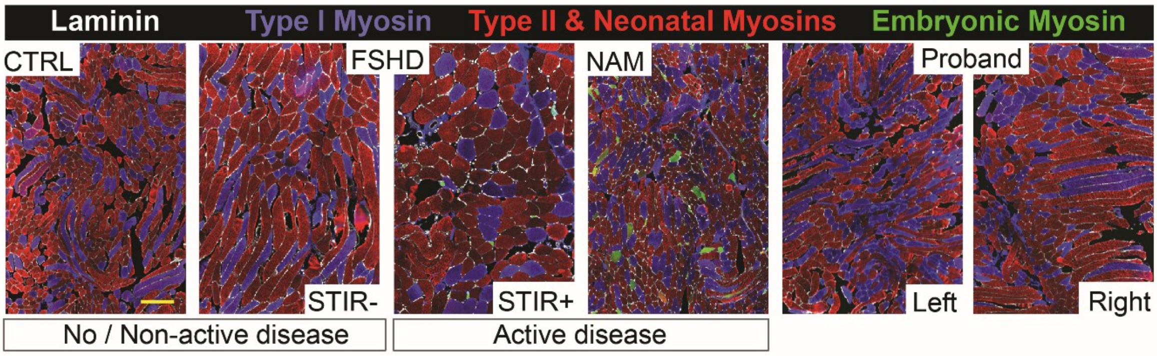

	% of Type I	% of Type II	% of I/II Hybrid	% Regenerative
**CTRL**	21.8	77.6	0.7	0.0
**FSHD_STIR-**	40.4	59.3	0.3	0.0
**FSHD_STIR+**	13.5	75.9	0.7	9.9
**NAM**	20.6	69.1	0.9	9.4
**Proband Left**	44.7	55.2	0.1	0.1
**Proband Right**	41.4	57.7	0.7	0.3

Supplementary Figure 3. **Proband biopsy displays a reduced fraction of regenerating fibres compared to FSHD-STIR+ and NAM.**

Representative images of immunolabelling for Slow (blue), Fast and Neonatal (red), and Embryonic myosin heavy chain (MYH3, green), together with Laminin (white), to highlight fibre types and fibres boundaries in the different biopsies examined, with fibre type distribution reported.

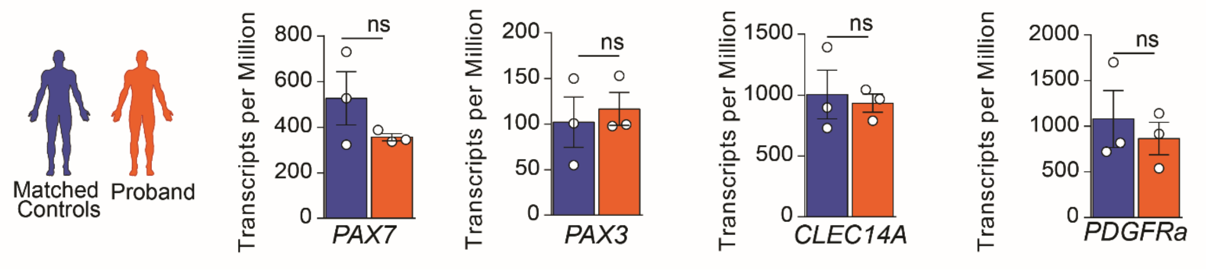

Supplementary Figure 4. **Transcriptomic analysis suggests no activation of compensatory pathways for muscle regeneration in the proband muscle.** Transcriptomic analysis shows no significant changes in indicated genes in proband muscle biopsies compared to controls.

Supplementary Table 1. **Quantitative muscle testing assessment on Proband**

Supplementary Table 2. **List of genes assessed on WES.**

Supplementary Table 3. **Information on control muscle specimens.**

Supplementary Table 4. **List of Differentially Expressed Genes**

Supplementary Table 5. **Gene Ontology (GO) analysis on DEG.**

Supplementary Table 6. **List of PAX7 variants reported with muscular or non-muscular phenotypes.**

Supplementary Table 7. **List of primers used in this study.**
